# *Borrelia burgdorferi *sensu lato in *Ixodes ricinus *ticks collected from migratory birds in Southern Norway

**DOI:** 10.1186/1751-0147-52-59

**Published:** 2010-11-06

**Authors:** Vivian Kjelland, Snorre Stuen, Tone Skarpaas, Audun Slettan

**Affiliations:** 1 University of Agder, Kristiansand, Norway; 2 National Veterinary Institute, Sandnes, Norway; 3 Sørlandet Hospital Health Enterprise (SSHF), Kristiansand, Norway

## Abstract

**Background:**

*Borrelia burgdorferi *sensu lato (s.l.) are the causative agent for Lyme borreliosis (LB), the most common tick-borne disease in the northern hemisphere. Birds are considered important in the global dispersal of ticks and tick-borne pathogens through their migration. The present study is the first description of *B. burgdorferi *prevalence and genotypes in *Ixodes ricinus *ticks feeding on birds during spring and autumn migration in Norway.

**Methods:**

6538 migratory birds were captured and examined for ticks at Lista Bird Observatory during the spring and the autumn migration in 2008. 822 immature *I. ricinus *ticks were collected from 215 infested birds. Ticks were investigated for infection with *B. burgdorferi *s.l. by real-time PCR amplification of the 16S rRNA gene, and *B. burgdorferi *s.l. were thereafter genotyped by melting curve analysis after real-time PCR amplification of the *hbb *gene, or by direct sequencing of the PCR amplicon generated from the *rrs *(16S)-*rrl *(23S) intergenetic spacer.

**Results:**

*B. burgdorferi *s.l. were detected in 4.4% of the ticks. The most prevalent *B. burgdorferi *genospecies identified were *B. garinii *(77.8%), followed by *B.valaisiana *(11.1%), *B. afzelii *(8.3%) and *B. burgdorferi *sensu stricto (2.8%).

**Conclusion:**

Infection rate in ticks and genospecies composition were similar in spring and autumn migration, however, the prevalence of ticks on birds was higher during spring migration. The study supports the notion that birds are important in the dispersal of ticks, and that they may be partly responsible for the heterogeneous distribution of *B. burgdorferi *s.l. in Europe.

## Background

The main vector for *Borrelia burgdorferi *sensu lato (s.l.) in Norway is the tick *Ixodes ricinus*. This tick is distributed along the coastal areas from Østfold in the south to Nordland in the north. Over the past decades, a remarkable increase in the density of tick populations in many areas of Norway, especially on islands, has been reported [1]. This may be due to factors as climatic changes, increased roe deer abundance and changes in habitat structure [2]. Birds, especially ground feeding species, are at risk of tick infestation, and are considered important in the global dispersal of ticks and tick-borne pathogens through their migration within and between continents [3-5]. In addition to transferring infected ticks, some avian species may also transport *B. burgdorferi *as an active infection [5,6]. Migratory birds have been shown to carry large amounts of ticks to Norway [7,8]. It is difficult to determine the place of origin of these ticks, and thereby the *B. burgdorferi *strains, due to the different origin and migratory route for different bird species, but the dominant direction of migration is from southwest to northeast during spring, and the opposite direction during autumn [9]. Although similar studies have been performed in other Nordic countries [3,4], this is the first description of *B. burgdorferi *prevalence and genotypes in *I. ricinus *ticks feeding on birds during spring and autumn migration in Norway.

The aim of this study was to contribute to the knowledge of migratory birds' involvement in the ecology of *B. burgdorferi *s.l. in Norway.

## Material and methods

### Bird capture and tick collection

Birds were trapped for ringing at Lista Bird Observatory in Southern Norway (58°06'N; 06°34'E) in the periods April - June and July - November 2008, periods that are representative of spring and fall migration. Due to a very high number of birds caught at some days during autumn migration, the Bird Observatory staff reported that the birds were not thoroughly examined for ticks every day. Surveillance of migratory birds by standardised trapping and ringing are the primary tasks for the bird observatory, however as some bird species are resident birds in the area, our material includes migratory as well as resident bird species. The birds were trapped in mist nets at ground level. After capture, the birds were identified to species, and their sex and age were determined if possible. Any ticks found on the head of the birds were removed and placed into plastic vials containing 70% ethanol. The ticks were stored at 4°C until further analysis. All ticks were identified to species according to Hillyard [10]. In addition, host-seeking ticks were sampled by flagging the undergrowth with a flannel cloth. The tick collection was performed during dry weather conditions (sunny/cloudy, no precipitation).

### DNA extraction

DNA was isolated by phenol/chloroform extraction. Briefly, nymphal and larval *I. ricinus *ticks were cleansed in phosphate buffered saline (PBS), then in sterile water, before being lightly dried of at a tissue paper. Nymphs were cut longitudinally in two halves using a sterile blade. The ticks were enzymatic digested overnight at 56°C in 180 μl lysis buffer (NaCl 0.1M, Tris-HCl 0.2M, pH 8.0 EDTA 0.05M, SDS 0.5%) and 20 μl proteinase K (20 mg/ml), followed by extraction using phenol/chloroform [11]. The DNA was precipitated with 1/10 volume NaAc 3M, pH 5.0 and 2.5 volume absolute ethanol, and resuspended in 200 μl 0.5 × TE buffer (Tris 5 mM, EDTA 0.5 mM, pH 8.0). Purified DNA was stored at - 20°C.

### Detection of *B. burgdorferi *s.l

DNA extracts were examined for *B. burgdorferi *spirochetes by using a real-time PCR assay with probe and primers specific for a section of the 16S rRNA gene [12] (Table 1). Real-time PCR was performed using iCycler/MyIQ™ (Bio-Rad, California, USA). Briefly, the 25 μl PCR mixture included 1X ready-to-use reaction mixture (TaqMan Universal PCR Master Mix, Applied Biosystems Inc., New Jersey, USA) containing reaction buffer, Taq DNA polymerase, deoxynucleoside triphosphate and MgCl_2_. The final concentration of the primers and probe were 1.125 μM and 0.25 μM, respectively. Finally, 5 μl of template DNA was added. The PCR conditions were as follows: 50°C for 2 min and 95°C for 10 min, followed by 50 cycles of 95°C for 15 s and 63°C for 60 s.

**Table 1:** Sequences for probes and primers used in this study.

	Sequence (5' - 3')	Reference
LB probe	6FAM-TTCGGTACTAACTTTTAGTTAA-MGBNFQ	[12]
LB forward primer	GCTGTAAACGATGCACACTTGGT	[12]
LB reverse primer	GGCGGCACACTTAACACGTTAG	[12]
Hbb probe	FAM-CAATGTCTGACTTAGTAACCTTTGGTCTTCTTGA-BHQ1	[13]
Hbb forward primer	GTAAGGAAATTAGTTTATGTCTTT	[13]
Hbb reverse primer	TAAGCTCTTCAAAAAAAGCATCTA	[13]
IGS 1 forward primer	GTATGTTTAGTGAGGGGGGTG	[14]
IGS 1 reverse primer	GGATCATAGCTCAGGTGGTTAG	[14]
IGS 2 forward primer	AGGGGGGTGAAGTCGTAACAAG	[14]
IGS 2 reverse primer	GTCTGATAAACCTGAGGTCGGA	[14]

### Genotyping *B. burgdorferi *species

Differentiation of the *B. burgdorferi *s.l. strains was done by a species-specific, single-run, real-time PCR based on the *hbb *gene sequence as previously described [13]. Briefly, real-time PCR was performed using iCycler/MyIQ™ (Bio-Rad). The 25 μl PCR mixture included 1X ready-to-use reaction mixture (TaqMan Universal PCR Master Mix). The final concentration of the primers and probe (Table 1) was 0.2 μM each. Finally, 5 μl of template DNA was added. The PCR conditions were as follows: 95°C for 10 min, followed by 55 cycles of 95°C for 30 s, 50°C for 45 s and 72°C for 30 s. The amplification was followed by a melting program, which started with denaturation at 95°C for 1 min, annealing at 35°C for 1 min, followed by 0.5°C temperature increase every 30 s until 85°C. During the slow heating process, fluorescence was measured at every 0.5°C. The melting points of the amplicons generated from the unknown samples and from known *B. burgdorferi *species were compared for genotyping.

In cases where melting temperature was in a region where a clear identification could not be made, genotyping was done by direct sequencing of the chromosome located *rrs *(16S)-*rrlA *(23S) intergenic spacer (IGS) [14]. See Table 1 for sequences for IGS primers. Briefly, the locus was amplified by a nested PCR procedure, comprising 35 cycles for the first reaction (IGS1) and 39 cycles for the second reaction (IGS2). The reaction conditions used were as follows: 95°C for 5 min, 94°C for 30 s, 50°C for the first reaction and 59°C for the second reaction for 30 s, and 74°C for 3 min. PCR products were sequenced directly in reverse direction on a 3130 Genetic Analyzer automated capillary sequencer (Applied Biosystems Inc.).

### Statistical analyses

Differences in the prevalence of *B. burgdorferi *s.l. in the larval and nymphal *I. ricinus *ticks collected during spring and autumn migration, respectively, were examined using χ2 test. Calculations were performed using SPSS statistical software, version 17. A probability of *P *< 0.05 was regarded as statistically significant.

## Results

### Tick infestation of birds

A total of 6538 birds of 85 species were captured and examined for ticks at Lista Bird Observatory during the spring and the autumn migration in 2008. Only *I. ricinus *ticks were found. 822 ticks were collected from 215 infested birds of 34 species, giving a prevalence of 3.3% (215 of 6538 birds), a relative intensity of 0.13 tick per bird (822 ticks per 6538 birds), and a mean intensity of 3.82 ticks per infested bird (822 ticks per 215 birds). The prevalence of infested birds were higher during spring migration compared to autumn migration, with 6.18% (64 of 1035 birds) and 2.74% (151 of 5503 birds), respectively. Furthermore, the relative intensity of tick infestation was also higher during spring migration compared to autumn migration, with 0.20 tick per bird (202 ticks per 1035 birds) and 0.11 tick per bird (620 ticks per 5503 birds), respectively. However, the mean intensity of tick infestation was lower during spring migration compared to autumn migration, with 3.2 ticks per infested bird (202 ticks per 64 birds) and 4.1 ticks per infested bird (620 ticks per 151 bird), respectively.

A total of 499 larvae and 323 nymphs were collected. No adult ticks were found. During spring migration, 53 larvae and 149 nymphs were collected (Table 2), whereas during autumn migration, 446 larvae and 174 nymphs were collected (Table 3). The bird species most commonly infested by ticks were tree pipit (*Anthus trivialis*) (36.9%, 7/19), chaffinch (*Fringilla coelebs*) (18.8%, 51/272), whitethroat (*Sylvia communis*) (16.5%, 20/121), dunnock (*Prunella modularis*) (15.6%, 5/32), lesser whitethroat (*Sylvia curruca*) (9.4%, 3/32), blackbird (*Turdus merula*) (9.2%, 22/238), song thrush (*Turdus philomelos*) (7.7%, 5/65), European robin (*Erithacus rubecula*) (7.1%, 29/411) and fieldfare (*Turdus pilaris*) (6.5%, 5/77).

**Table 2:** Tick infestation of birds and *B. burgdorferi *s.l. prevalence in *I. ricinus*, spring 2008.

Bird species	No. birds	No. ticks	No. (%) birds infested	Mean no. ticks per infested bird	*Borrelia *infected larvae/no. larvae examined	*Borrelia *infected nymphs/no. nymphs examined
**Migrating birds**						
*Acrocephalus arundinaceus*	1	1	1 (100)	1		0/1
*Acrocephalus palustris*	5	1	1 (20)	1		0/1
*Acrocephalus scirpaceus*	3	1	1 (33.3)	1		0/1
*Sylvia borin*	17	1	1 (5.9)	1		0/1
*Sylvia communis*	28	5	3 (10.7)	1.7	0/1	0/4
*Luscinia svecica*	1	1	1 (100)	1	0/1	
*Carduelis cannabina*	19	1	1 (5.3)	1		0/1
*Carduelis cabaret*	1	1	1 (100)	1	0/1	
*Carpodacus erythrinus*	4	1	1 (25)	1		0/1
*Coccothraustes coccothraustes*	1	2	1 (100)	2		0/2
Phylloscopus collybita*	65	1	1 (1.5)	1		0/1
Sylvia atricapilla*	43	1	1 (2.3)	1		0/1
Erithacus rubecula*	232	49	24 (10.3)	2	0/27	1/22
Turdus iliacus*	4	8	1 (25)	8	0/1	2/7
Turdus merula*	77	83	12 (15.6)	6.9	0/14	2/69
Turdus pilaris*	29	4	3 (10.3)	1.3		1/4
Turdus philomelos*	17	14	2 (11.8)	7	0/3	0/11
Fringilla coelebs*	14	2	1 (7.1)	2	0/1	0/1
Prunella modularis*	32	23	5 (15.6)	4.6	0/3	2/20
Sturnus vulgaris*	16	1	1 (6.3)	1	0/1	
**Resident birds**						
*Carduelis carduelis*	5	1	1 (20)	1		0/1
**Total**	**614**	**202**	**64 (10.4)**	**3.2**	**0/53**	**8/149**

Migrating and resident birds are defined according to Fonstad et al. [9]. Only bird species with at least one tick infested individual are included in the table.

*Migrating birds, but some individuals may overwinter

**Table 3:** Tick infestation of birds and *B. burgdorferi *s.l. prevalence in *I. ricinus*, autumn 2008.

Bird species	No. birds	No. ticks	No. (%) birds infested	Mean no. ticks per infested bird	*Borrelia *infected larvae/no. larvae examined	*Borrelia *infected nymphs/no. nymphs examined
**Migrating birds**						
*Acrocephalus scirpaceus*	5	1	1 (20.0)	1	0/1	
*Phylloscopus trochilus*	440	44	24 (5.5)	1.8	0/21	1/23
*Sylvia curruca*	32	4	3 (9.4)	1.3	0/3	0/1
*Sylvia communis*	93	49	17 (18.3)	2.9	1/31	0/18
*Motacilla flava*	3	1	1 (33.3)	1		0/1
*Oenanthe oenanthe*	99	1	1 (1.0)	1		0/1
*Carduelis cabaret*	8	4	2 (25)	2	0/2	0/2
*Carduelis cannabina*	41	1	1 (2.4)	1	0/1	
*Anthus trivialis*	19	18	7 (36.9)	2.6	0/8	1/10
Sylvia atricapilla*	135	15	6 (4.4)	2.5	0/10	0/5
Erithacus rubecula*	179	14	5 (2.8)	2.8	0/10	0/4
Turdus iliacus*	64	16	2 (3.1)	8	3/4	7/12
Turdus pilaris*	48	7	2 (4.2)	3.5		0/7
Turdus philomelos*	48	17	3 (6.3)	5.7	0/9	0/8
Turdus merula*	161	44	10 (6.2)	4.4	3/12	2/32
Emberiza schoeniclus*	26	3	2 (7.7)	1.5	0/1	0/2
Fringilla coelebs*	258	361	50 (19.4)	7.2	7/322	2/39
Fringilla montifringilla*	48	3	1 (2.1)	3	0/2	0/1
Anthus pratensis*	59	2	2 (3.4)	1	0/1	0/1
**Resident birds**						
*Cyanistes caeruleus*	1325	6	4 (0.3)	1.5	0/1	0/5
*Lophophanes cristatus*	52	1	1 (1.9)	1		0/1
*Parus major*	147	1	1 (0.7)	1	0/1	
*Carduelis chloris*	99	1	1 (1.0)	1	0/1	
*Troglodytes troglodytes*	150	6	4 (2.7)	1.5	1/5	0/1
**Total**	**3539**	**620**	**151 (4.3)**	**4.1**	**15/446**	**13/174**

Migrating and resident birds are defined according to Fonstad et al. [9]. Only bird species with at least one tick infested individual are included in the table.

*Migrating birds, but some individuals may overwinter

### Host-seeking ticks

Migrating birds may transport ticks over long distances, but tick infestation may also be a result of local tick recruitment. To investigate the potential role of local tick recruitment, host-seeking ticks were collected in the vicinity of the bird observatory. Five hours of flagging yielded only 6 *I. ricinus *ticks in areas within 0.5 km from the bird observatory. However, flagging of sites approximately 4 km and 13 km from the observatory were performed in the same period, and yielded 428 (173 nymphs and 255 larvae) and 298 (5 adults, 154 nymphs, 140 larvae) ticks per hour of flagging, respectively. *I. ricinus *ticks collected at the site 13 km from the observatory were examined for *B. burgdorferi *s.l., and the spirochetes were detected in 24.1% of nymphal and 16.7% of adult ticks [13].

### *B. burgdorferi *s.l. infection of ticks and genospecies identification

*B. burgdorferi *s.l. were detected in 4.4% of the ticks. In ticks collected during spring migration, *B. burgdorferi *s.l. were detected in 5.4% of nymphal ticks (Table 2), whereas in ticks collected during autumn migration, the spirochetes were detected in 3.4% of larvae and 7.5% of nymphs (Table 3). The differences in *B. burgdorferi *s.l. prevalence in larva and nymphs were not statistically significant between spring and autumn migration. *B. garinii *was detected in 77.8%, *B. valaisiana *in 11.1%, *B. afzelii *in 8.3% and *B. burgdorferi *sensu stricto (s.s.) in 2.8% of the ticks (Table 4). Mixed infections with more than one genospecies were not detected in any ticks.

**Table 4:** *B. burgdorferi *s.l. genotypes in *I. ricinus *ticks collected from birds, 2008.

		*Borrelia *species identified in larvae			*Borrelia *species identified in nymphs			
Bird species	No. birds with infected ticks/no. birds infested	Bg	Bv	Ba	Bg	Bv	Ba	Bbss
**Migrating birds**								
*Phylloscopus trochilus*	1 (24)							1
*Sylvia communis*	1 (20)	1						
*Anthus trivialis*	1 (7)				1			
Erithacus rubecula*	1 (29)						1	
Turdus iliacus*	2 (3)	3			9			
Turdus merula*	4 (22)	1	2		2	2		
Turdus pilaris*	1 (5)				1			
Fringilla coelebs*	8 (51)	7			2			
Prunella modularis*	2 (5)				1		1	
**Resident birds**								
*Troglodytes troglodytes*	1 (4)			1				
**Total**	**22 (170)**	**12**	**2**	**1**	**16**	**2**	**2**	**1**

Migrating and resident birds are defined according to Fonstad et al. [9]. Only bird species carrying *B. burgdorferi *s.l. infected tick(s) are included in the table.

*Migrating birds, but some individuals may overwinter

## Discussion

During spring and autumn migration 2008, 6538 birds were examined for tick infestation at Lista Bird Observatory in Southern Norway, and 822 immature *I. ricinus *were collected from 215 birds. Large variations were found in the contributions of the different bird species to the number of ticks collected. Ticks were found on 34 of the 85 bird species examined. The bird species most commonly infested by ticks were *Turdus *spp., *Anthus trivialis*, *Fringilla coelebs*, *Sylvia spp*., *Prunella modularis *and *Erithacus rubecula*, consistent with previous Norwegian studies [7,8,15,16]. These avian species are ground-feeding, which puts them at risk of tick infestation.

The prevalence of ticks on the birds was higher during spring migration compared to autumn migration, with 6.2% and 2.7% of the birds infested, respectively. This may be explained by different tick activity during spring and autumn migration and/or by different tick population densities along the birds' migration routes in to Norway (spring) compared to in their Norwegian breeding grounds (autumn). However, it is possible that the observed difference is due to the much higher number of birds caught during autumn migration compared to spring migration (5503 and 1135 birds, respectively), which left less time for the bird observatory staff to examine each bird for ticks. However, the mean intensity of tick infestation was higher during autumn migration compared to spring migration, with 4.1 and 3.2 ticks per infested bird, respectively. The reason for this is unknown.

Few ticks were found in the immediate distance (<0.5 km) from the bird observatory, however, high *I. ricinus *densities were found in other sites in the region, and local tick recruitment cannot be excluded. Future studies should investigate this, for example by studying genetic variation in *I. ricinus *ticks along migratory routes as previously described [17].

The prevalence of *B. burgdorferi *in ticks collected from birds was 4.4% (4.0% and 4.5% in ticks collected during spring and autumn migration, respectively). In ticks collected during spring migration, *B. burgdorferi *s.l. were detected in 5.4% of nymphal ticks, whereas in ticks collected during autumn migration, the spirochetes were detected in 3.4% of larvae and 7.5% of nymphs, however, these differences were not statistically significant. The most prevalent genospecies were *B. garinii *(77.8%), followed by *B. valaisiana *(11.1%), *B. afzelii *(8.3%) and *B. burgdorferi *s.s. (2.8%). Other Nordic studies have reported findings of similar infection rate and genospecies composition in ticks collected from migrating birds [3,4]. *B. burgdorferi *genospecies composition in host-seeking ticks in Southern Norway was described in a previous study [13]. Although local variations were observed, the overall prevalence of *B. burgdorferi *s.l. in *I. ricinus *was 22.3%, and the general pattern was a dominance of *B. afzelii*, followed by *B. garinii*, *B. burgdorferi *s.s, and *B. valaisiana*. The low prevalence of *B. burgdorferi *s.l. in ticks collected from birds compared to host-seeking ticks may be explained by the observed differences in sensitivity to host serum among the *B. burgdorferi *s.l. strains. During feeding, ticks take up host-derived molecules as complement and other blood components. It has been proposed that the genospecies *B. afzelii *is sensitive to avian complement, and that these spirochetes are eliminated in the tick midgut, whereas *B. garinii *survives such a blood meal and can be transmitted to the host [18]. Comparison with present finding of genospecies composition in ticks feeding on birds support the notion of a genospecies specific association between birds and *B. garinii*, and an elimination of *B. afzelii *infections.

*B. burgdorferi *infection was detected in 3.8% of larvae carried by the avian species redwing (*Turdus iliacus*), blackbird (*Turdus merula*), chaffinch (*Fringilla coelebs*), whitethroat (*Sylvia communis*) and winter wren (*Troglodytes troglodytes*). Larval infection does not necessarily imply host reservoir competence, as infection may also arise from transovarial transmission or from co-infection [19]. However, previous studies have demonstrated *Turdus *spp. as reservoir hosts for *B. garinii *and *B. valaisiana *[6,20,21], supporting the possibility of the bird as a source of infection. However, further studies are necessary to determine the potential reservoir capacity of chaffinch, whitethroat and winter wren.

As previously described, the birds were not thoroughly examined for ticks every day during autumn migration. Furthermore, our material includes migratory as well as resident bird species. These factors may have influenced findings in the present study, and future studies should attempt to avoid these potentially confounding factors.

## Conclusion

These data support the notion that birds may be partly responsible for the heterogenous distribution of *B. burgdorferi *s.l. in Europe. Further studies are necessary to evaluate the impact of different bird species on Lyme borreliosis ecology. Ticks may be infected by a wide range of important pathogens, including tick-borne encephalitis virus (TBEV) and *Anaplasma phagocytophilum*, and future studies should also include investigation of the birds' role in the ecology of these pathogens.

## Competing interests

The authors declare that they have no competing interests.

## Authors' contributions

VK, SS, TS and AS designed the study. VK carried out the experiments. VK and SS drafted the manuscript. AS provided technical assistance. All authors read and approved the final manuscript.
